# Severity and Influencing Factors of Homophobia in Korean Nursing Students

**DOI:** 10.3390/ijerph16234692

**Published:** 2019-11-25

**Authors:** Hye Weon Kwak, Min Young Kim, Min Young Kim

**Affiliations:** 1 Department of Nursing, Daekyeung University, Gyeongsan 38547, Korea; hwk@tk.ac.kr (H.W.K.); bubulo@tk.ac.kr (M.Y.K.); 2 Department of Nursing, Ulsan University, Ulsan 44610, Korea

**Keywords:** homophobia, nursing students, factors, sexual minorities

## Abstract

Sexual minorities are people with non-cis and non-heterosexual gender identities, including LGBT (lesbian, gay, bisexual, and transgender) identities. Korean society is prejudiced against sexual minorities—in our study, we will broadly label this prejudice homophobia. It is possible that sexual minorities do not receive appropriate health management owing to such prejudices. Therefore, it is necessary to reduce homophobia in nursing students. This study aims to measure the degree of homophobia in Korean nursing students and identify the factors that affect homophobia. Our study is a cross-sectional study, which surveys attitudes of 265 nursing students toward homophobia in five Korean cities in January to March, 2019. The average homophobia score was 74.5 out of a possible 120; 92.9% of the participants were classified as homophobic, and 42.3% as highly homophobic. We found that participants who were male, religious, had low self-esteem, and had no family members or acquaintances who might belong to a sexual minority group, were more likely to be homophobic. Nursing students in Korea still exhibit high levels of homophobia. As high levels of homophobia can negatively affect health management and nursing, especially in the case of sexual minorities, we suggest that educational programs should be set up to reduce homophobia in nursing students.

## 1. Introduction

The term ‘sexual minority’ refers to individuals with non-cis, non-heterosexual gender identities. Such minorities include LGBT (lesbian, gay, bisexual, and transgender), intermediate sex, genderqueer, and third sex individuals [1]. Recently, Korean society has become more interested in sexual minorities, who are changing widespread perceptions of themselves by holding the Queer Culture Festival. Perceptions of sexual minorities have improved and public awareness has increased; however, according to a 2017 survey, 58.0% of Koreans said they could not accept sexual minorities [2]. Prejudice against sexual minorities clearly still exists in Korean society.

Prejudice against sexual minorities is referred to as homophobia. It encompasses negative attitudes and emotions, such as contempt, hatred, or antipathy for sexual minorities, which may be caused by irrational fear or ignorance [3,4]. Homophobia exists in various forms in society. Sexual minorities often experience unfair discrimination and are excluded from social activities because of homophobia [5,6]. Homophobia also negatively affects the health activities of sexual minorities [7]. For example, some individuals from sexual minorities hesitate to visit medical institutions, and therefore their health problems remain untreated [8,9]. In a previous study, some nurses expressed that they fear and reject sexual minorities because of homophobia, which led them to engage in inappropriate nursing behavior [3]. As many patients’ first point of contact when seeking clinical care, nurses should be unprejudiced, accept patients as they are, and attend to their needs. In addition, nursing students, as prospective nursing practitioners required to nurse all and any patients, will need to develop nursing service plans in the future based on experience with and awareness of sexual minorities. These kinds of prejudices, even when recognized, are not easily eradicated. Therefore, it is necessary to provide nurses with robust, relevant education from the time they are students. One study reported that more than 79% of nursing teachers said it was important to teach nursing students about homophobia [10]. Furthermore, studies have shown that homophobia in nursing college students decreases after attending sexual minority-related education programs [11].

Despite the need for these education programs, research on sexual minorities in Korea is lacking. This research is generally limited to research on the stress felt by sexual minorities [12] and research on sexual minorities related to social prejudice [13]. There are a few studies involving nurses who are responsible for the health of sexual minorities [14]. However, there are no studies regarding sexual minorities and Korean nursing students. Therefore, the objective of this study is to investigate homophobia in Korean nursing students and the factors affecting their homophobia.

## 2. Materials and Methods

### 2.1. Study Design

This was a descriptive, cross-sectional study designed to investigate the effects of homophobia in Korean nursing students.

### 2.2. Sample and Setting

Participants were 265 nursing students who understood the purpose of the study and voluntarily agreed to participate; they were third- and fourth-grade students in nursing schools in five regions across the country.

A minimum sample size of 200 was calculated using the G-power 3.1 program with a significance level of 0.05 for multiple regression analysis, a middle effect size of 0.15, and a power of 80%. Thus, we recruited 265 individuals to allow for incomplete questionnaires.

### 2.3. Data Collection

Data were collected using structured questionnaires over a total of 11 days, from January to March, 2019. After explaining the purpose of the study to the head of the nursing department at each participating university, we put up posters at these universities to inform prospective participants about the study in advance. We then visited classes at an appropriate time to explain the study and obtain consent. Students who agreed to participate in the study were required to complete the questionnaire. A total of 270 questionnaires were distributed and 265 were collected and analyzed (a 98% recovery rate).

### 2.4. Instruments

#### 2.4.1. Homophobia

We used the Korean version of the Index of Homophobia developed by Hudson and Rickets [4] as adapted by Kim and Bahn [15] to measure nursing students’ negative attitudes toward homosexuality. Items were rated on a 5-point Likert scale from 1 (strongly disagree) to 5 (strongly agree), and 13 of the 24 questions were scored in reverse. The total score ranges from 24 to 120. A score of 76 or higher is considered a highly homophobic response, a score of 51 to 75 a lower homophobic response, a score of 26 to 50 a low non-homophobic response, and a score of 24 to 25 a highly non-homophobic response. At the time of development, the tool had a Cronbach’s alpha of 0.94; in this study it was 0.94.

#### 2.4.2. Participant Characteristics

General characteristics included gender, age, whether practicing a religion, irritable personality (becoming annoyed or angry very easily), importance placed on interpersonal relationships, self-esteem and clinical practice stress. Irritable personality and importance placed on interpersonal relationships were self-reported. Sexual minority-related characteristics included educational experience regarding sexual minorities, whether or not a family member or acquaintance identified as a sexual minority, and experience with sexual minorities in other environments.

Self-esteem was assessed via the Rosenberg Self-Esteem Scale [16], which is designed to measure self-esteem and consists of 10 items rated on a 4-point Likert scale from 1 (strongly disagree) to 4 (strongly agree). The total score ranges from 10 to 40, with higher scores indicating higher self-esteem. At the time of scale development, Cronbach’s alpha was 0.85; in this study, it was 0.81.

Clinical practice stress was assessed with the Stress Inventory developed by Beck and Srivastava [17]. We used 24 statements measuring clinical practice stress, and items are rated on a 5-point Likert scale from 1 (not at all) to 5 (very much). The total score ranges from 24 to 120, with higher scores indicating higher levels of stress. At the time of scale development, Cronbach’s alpha was 0.84; in this study, it was 0.88.

### 2.5. Ethical Considerations

This study was approved by the Korea National Institute for Bioethics (Approval No.: P01-201812-22-006). In order to protect the rights of the study participants, the purpose and method of the study and the study’s positions on participants’ privacy and confidentiality, voluntary consent, and possible withdrawal were explained to the study participants before collecting the data. All participants provided written informed consent.

### 2.6. Statistical Analysis 

Data analyses were performed using SPSS 20.0 (IBM SPSS Inc., Chicago, IL, USA). Participants’ general characteristics and homophobia scores were analyzed using descriptive statistics such as frequency, percentage, mean, and standard deviation. Differences in homophobia scores according to general characteristics were analyzed with Mann-Whitney U tests. To identify the predictive factors of homophobia in Korean nursing students, a multiple linear regression analysis was conducted.

## 3. Results

Baseline general characteristics of our study sample are displayed in Table 1. The average age of participants was 23.0 years and gender of participants was women 225 (84.9%). A total of 169 participants (63.8%) had no religion and 118 (70.9%) reported irritable personality, while 223 (87.9%) answered importance of interpersonal relationships. The mean self-esteem score was 31.8 ± 4.5 over 40 points and the mean score of clinical practice stress was 75.6 ± 13.1 over 120 points. A total of 56 participants (21.1%) had educational experience of sexual minorities, 38 (14.3%) had family members or acquaintances who identified as a sexual minority, and 31 (11.7%) had experience with sexual minorities in some other environment.

### 3.1. Level of Homophobia

**Figure 1:**
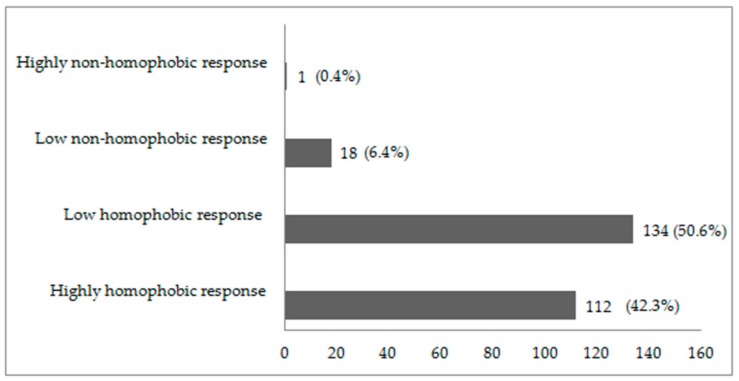
Participants’ levels of homophobia.

A total of 112 participants (42.3%) had highly homophobic responses, while 134 (50.6%) had low homophobic responses. Only 1 (0.4%) participant had a highly non-homophobic response (Figure 1).

### 3.2. Items of Homophobia Scale

The items of this study’s homophobia scale are listed in Table 2. The mean homophobia score was 74.5 ± 17.1 over 120 points. In our study, the statements, “I would feel comfortable if a member of my sex made an advance toward me” and “I would enjoy attending social functions at which homosexuals were present” (these questions were scored in reverse) represented the highest homophobia scores, and the statements, “It would disturb me to find out that my doctor was homosexual” and “I would feel uncomfortable if I learned that my neighbor was a homosexual” represented the lowest homophobia scores.

### 3.3. Homophobia Score According to General Characteristics

Differences in participants’ homophobia scores according to participants’ characteristics and experience with sexual minorities are shown in Table 3. Participants’ homophobia was significantly different according to gender, religion and whether the participant had family members or acquaintances who identify as a sexual minority: females and those who had family members or acquaintances who identify as a sexual minority showed lower homophobia.

### 3.4. Factors Affecting Homophobia

Multivariate analyses revealed which variables affect participants’ level of homophobia (Table 4). Variables that affected homophobia were gender: female (B = −15.81, *p* = 0.001), religion (B = 5.74, *p* = 0.004), having family members or acquaintances who identify as a sexual minority (B = −9.26, *p* = 0.001), and self-esteem (B = −0.67, *p* = 0.002). 

## 4. Discussion

This study aimed to both identify the level of homophobia in Korean nursing students in Korea and identify the factors that affect homophobia in Korean nursing students.

The average homophobia score in this study was 74.5; 92.9% of the participants can be classified as homophobic, and 42.3% as highly homophobic. According to the literature, this means that rates of homophobia are higher in Korean nursing students than in nursing and medical students from most other countries—in the literature, between 7% and 40% of students in other countries could be classified as homophobic [18,19,20,21]. In addition, the results of a previous study showed that Asians have higher homophobia scores than do non-Hispanic Caucasians [22]. In addition, 78.9% of participants responded that they were not educated on sexual minorities and issues related to sexual minorities. According to a study by Cornelius and Carrick [23], when caregivers increased their knowledge and understanding of sexual minorities’ health management problems, they were more likely to care for patients identifying as a sexual minority. In addition, when there was education related to sexual minority-specific terminology, health, medical needs, and communication, caregivers demonstrated an improvement in their attitude towards sexual minorities [24,25]. We can conclude from these results that there is an urgent need to raise Korean nursing students’ awareness and tolerance of sexual minorities and related issues, perhaps through educational programs.

In this study, we found that factors which affect homophobia were being male, religion, not having a family member or acquaintance belonging to a sexual minority group, and low self-esteem. This is consistent with the results of prior studies, which showed that males exhibit higher and more intense rates of homophobia than females [20,26,27]. As Korean society is conservative and patriarchal, Korean men may have especially negative attitudes toward sexual minorities because Korean men are inclined to be more conservative. Religiosity’s effect on homophobia is also consistent with the results of prior studies. These studies have indicated that countries with highly Christian (especially Catholic) and Muslim populations have higher rates of homophobia [19,20,22,27,28]. 

In addition, participants who indicated that they have a family member or acquaintance who identifies as a sexual minority demonstrated lower rates of homophobia. This too is consistent with previous studies, which have indicated that homophobia is less likely among people who have lots of exposure to sexual minorities [22]. This shows that homophobia arises in part from vague, negative preconceptions. Therefore, educators aiming to reduce homophobia aim to facilitate contact and relationships between cis and non-cis groups of students to attempt to head off this kind of prejudice. 

Homophobia was also high in nursing students who reported low self-esteem. This is consistent with prior studies which indicate that low self-esteem individuals showcase less empathy and are generally more homophobic [27,29]. Self-esteem shows self-respect, and generally speaking, individuals with high self-esteem have greater respect for others. Individuals who show respect for others show their acceptance and recognition of others who are different from oneself [29]. In other words, education efforts should be aimed at both creating a space of empowerment and at embracing differences between individuals. Therefore, nursing students should improve their self-esteem to understand and accept the positions of the patients. There should be humanities and social education to improve the self-esteem of nursing students to allow them to understand others.

Although there is a change in awareness of sexual minorities through various efforts of society, Korea still has high homophobia and prejudices towards sexual minorities. Given nurses’ mandate to protect and care for patients’ lives, efforts must be undertaken to change nursing students’ attitudes on this front; sexual minorities should receive health services and be able to maintain their health without facing prejudice. Therefore, it is important to reduce homophobia in nurses, who are on the front lines of healthcare. 

This study is significant because it is the first study on homophobia in Korean nursing students. However, it has some limitations. First, because participants were students from five universities, other researchers should be cautious about generalizing the results of this study. Future studies should focus on university students all over the country. Second, this study identified fragmentary factors out of the general characteristics to analyze the correlation with homophobia. However, as homophobia can arise from many different factors and influences, future research should undertake more factor analysis by investigating various literature.

## 5. Conclusions

This study identified the level of homophobia in nursing students in Korea and the factors that affect homophobia. We found 92.9% of the participants were classified as homophobic, and 42.3% as highly homophobic; their mean homophobia score was 74.5 out of a possible 120. The study found higher rates of homophobia in participants who were male, religious, did not have a family member or acquaintance who identified as a sexual minority, and who had low self-esteem. Based on the results of this study, we suggest that health policy-makers implement education programs to mitigate against the formation and strengthening of these prejudices. 

## Figures and Tables

**Table 1:** General characteristics (*n* = 265).

Characteristics	Categories	*n* (%) or Mean ± SD *
Gender	Female Male	225 (84.9) 40 (15.1)
Age (years old)	20–30 >30	23.0 ± 3.0 261 (98.5) 4 (1.5)
Religion	Yes No	96 (36.2) 169 (63.8)
Irritable personality	Yes No	188 (70.9) 77 (29.1)
Importance of interpersonal relationships	Yes No	233 (87.9) 32 (12.1)
Self-esteem		31.8 ± 4.5
Clinical practice stress		75.6 ± 13.1
Educational experience of sexual minorities	Yes No	56 (21.1) 209 (78.9)
Family member or acquaintance identifying as sexual minority	Yes No	38 (14.3) 227 (85.7)
Experience with sexual minorities in some other environment	Yes No	31 (11.7) 234 (88.3)

* SD: Standard deviation.

**Table 2:** Items of the study’s homophobia scale.

Items	Score Mean ± SD *
7. I would feel comfortable if a member of my sex made an advance toward me.^1^	3.9 ± 0.9
2. I would enjoy attending social functions at which homosexuals were present.^1^	3.7 ± 0.9
8. I would be comfortable if I found myself attracted to a member of my sex.^1^	3.6 ± 1.0
17. I would feel comfortable if I learned that my spouse or partner was attracted to members of his or her sex.^1^	3.6 ± 0.9
6. I would feel uncomfortable being seen in a gay bar.	3.5 ± 1.1
23. I would feel comfortable if I learned that my son’s teacher was a gay.^1^	3.4 ± 1.0
5. I would feel comfortable knowing that I was attractive to members of my sex.^1^	3.4 ± 1.0
11. I would feel comfortable knowing that my co-worker was a homosexual.^1^	3.3 ± 0.9
19. I would feel comfortable if I learned that my boss was homosexual.^1^	3.2 ± 0.9
1. I would feel comfortable working closely with a male homosexual.^1^	3.2 ± 1.0
9. I would feel disappointed if I learned that my child was homosexual.	3.2 ± 1.2
15. If a member of my sex made an advance toward me, I would be offended.	3.2 ± 1.1
4. If a member of my sex made a sexual advance toward me I would feel angry.	3.1 ± 1.2
18. I would feel at ease talking with a homosexual person at a party.^1^	3.1 ± 0.8
12. I would be upset if I learned that my brother or sister was homosexual.	3.0 ± 1.2
20. I am not burdened by passing through the gay zone.^1^	3.0 ± 1.0
22. I would feel comfortable if I learned that my best friend of my sex was homosexual.^1^	3.0 ± 1.1
24. I would feel comfortable working closely with a female homosexual.^1^	2.8 ± 1.0
16. I would feel nervous if I learned that my daughter’s teacher was a lesbian.	2.7 ± 1.2
13. I would feel that I had failed as a parent if I learned that my child was gay.	2.7 ± 1.2
10. I would feel nervous being in a group of homosexuals.	2.7 ± 1.1
14. If I saw two men holding hands in public, I would feel disgusted.	2.4 ± 1.1
21. It would disturb me to find out that my doctor was homosexual.	2.3 ± 1.1
3. I would feel uncomfortable if I learned that my neighbor was a homosexual.	2.3 ± 1.0
**Total**	74.5 ± 17.1

* SD: Standard deviation. ^1^ Questions scored in reverse.

**Table 3:** Homophobia score according to general characteristics (*n* = 265).

Characteristics	Categories	Homophobia	Z	*p*
		Mean ± SD *		
Gender	Female Male	72.1 ± 16.2 88.1 ± 15.5	−5.29	<0.001
Age (years old)	20–30 >30	74.4 ± 17.1 81.5 ± 16.1	−0.90	0.366
Religion	Yes No	77.6 ± 16.0 72.7 ± 17.4	−2.02	0.043
Irritable personality	Yes No	75.4 ± 17.0 72.3 ± 17.0	−1.34	0.179 *
Importance of interpersonal relationships	Yes No	74.9 ± 17.7 71.3 ± 10.6	−1.43	0.153
Educational experience of sexual minorities	Yes No	73.1 ± 19.1 74.9 ± 16.5	−0.93	0.352
Family member or acquaintance identifying as sexual minority	Yes No	64.5 ± 17.9 76.2 ± 16.4	−3.76	<0.001
Experience with sexual minorities at university	Yes No	72.6 ± 11.8 74.7 ± 17.6	−0.54	0.589

* SD: Standard deviation.

**Table 4:** Factors affecting homophobia by multiple regression analysis (*n* = 265).

Variable	Category	B	SE	ß	t	*p*
Gender	Female	−15.81	2.66	−0.33	−5.95	<0.001
Religion	Yes	5.74	1.96	0.16	2.93	0.004
Family member or acquaintance identifying as a sexual minority	Yes	−9.26	2.71	−0.19	−3.42	0.001
Self-esteem		−0.67	0.21	-0.18	−3.17	0.002

B: the unstandardized beta; β: the standardized beta.
